# Importance of Genetic Diagnosis in Global Developmental Delay: A Case of Cabezas Syndrome Caused by CUL4B Gene Deletion and Not Identified by Array-CHG

**DOI:** 10.7759/cureus.46010

**Published:** 2023-09-26

**Authors:** Teresa L Magalhães, Mariana V Viegas, Catarina Mendonça, André Travessa, Daniel Soares

**Affiliations:** 1 Pediatrics, Centro Hospitalar do Oeste, Caldas da Rainha, Caldas da Rainha, PRT; 2 Genetics, Hospital Santa Maria, Lisboa, PRT

**Keywords:** intellectual disability (id), cabezas syndrome, pediatrics, genetic testing, developmental disabilities

## Abstract

Global developmental delay (GDD) and intellectual disability (ID) are common reasons for referral to neurodevelopmental assessment. The etiology of GDD and ID can be genetic, acquired, or multifactorial. We report a case of a 10-year-old boy with ID and GDD who was diagnosed with Cabezas syndrome, a rare genetic disorder caused by a deletion of the CUL4B gene. Despite normal results from previous testing, exome sequencing with copy number variation analysis led to the identification of the deletion. Early diagnosis of GDD and ID is crucial for effective patient management, including planning interventions and providing support, therapy, and genetic counseling for families.

## Introduction

Global developmental delay (GDD) and intellectual disability (ID) or their suspicion are common reasons for referral to neurodevelopmental assessments [1,2]. ID includes a group of clinically and genetically diverse disorders in which brain development and/or function are impaired. It can be classified as nonsyndromic or syndromic, depending on whether it is the only clinical feature or whether it is associated with other neurological and/or behavioral manifestations and structural anomalies [1-3].

The etiology of GDD and ID can be genetic, acquired, or multifactorial. In some cases, there may be no identifiable cause. A thorough medical history, physical examination, and specialized testing are required to determine the underlying cause of GDD and ID [1-4].

## Case presentation

**Figure 1:**
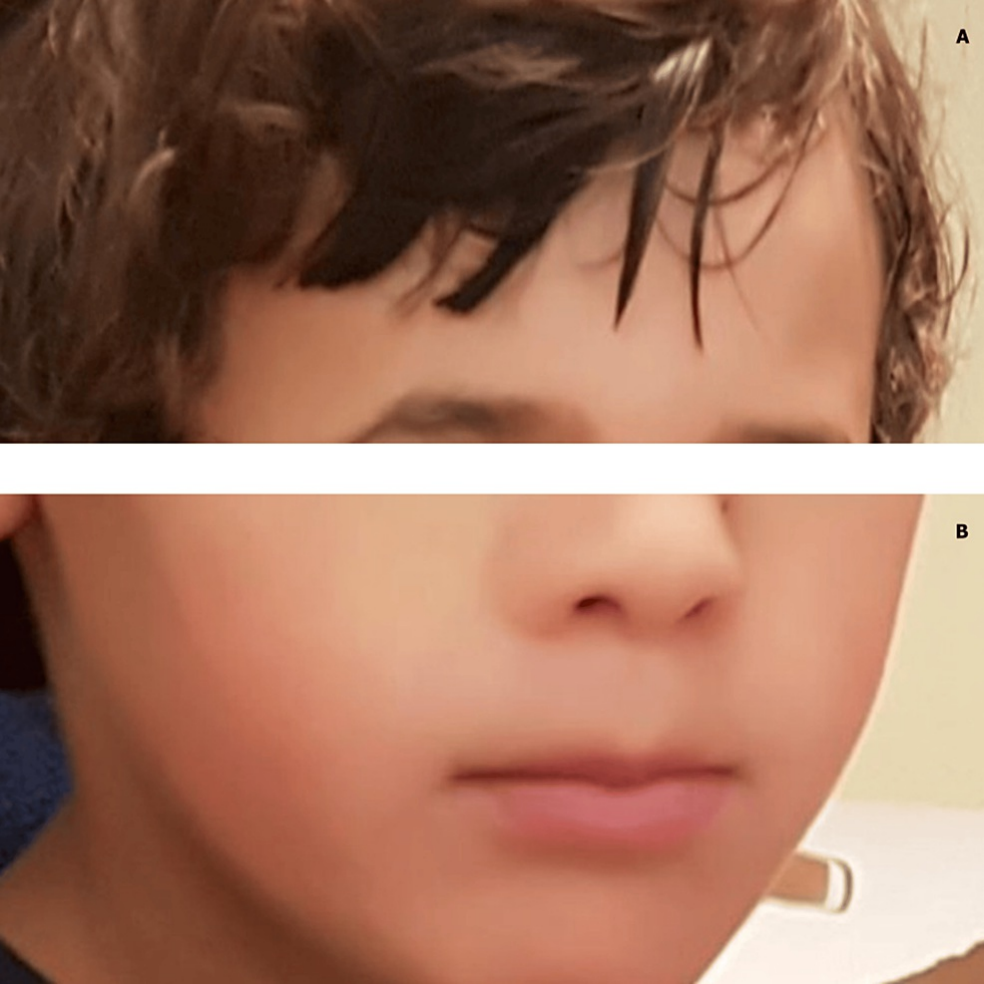
Phenotype of the boy assessed in medical consultation at five years of age The child's phenotype is shown during a clinical evaluation at five years of age. Written informed consent was obtained from the child's legal guardian for the publication of this photograph. This figure demonstrates the child's characteristic facial features, including a prominent forehead (A), a broad nasal bridge, and a thick lower lip (B), typical of Cabezas syndrome.

We present the case of a 10-year-old boy who was referred to neurodevelopmental assessment at the age of 24 months due to GDD, with a greater impact on the language area and poor height-weight progression. He is the firstborn child of healthy, non-consanguineous parents. The pregnancy was poorly supervised, and the child was delivered at 39 weeks and 5 days with a birth weight of 2220 g. No significant medical history was reported. The child's weight and height progression were below the 5th percentile. The child started kindergarten at the age of two and a half years, and the parents reported delayed psychomotor development. The child held his head up at six months, sat at 12 months, and crawled at 20 months. The child started walking at the age of two years. The child also had severe intellectual disability and pronounced language delay, and at the age of five, he was unable to speak a single word, despite apparently having good understanding. At 25 months, the Schedule of Growing Skills assessment revealed developmental delays in all areas between 12 and 18 months. He was referred to a local team for early intervention, educational support, occupational and speech therapy, and psychomotor rehabilitation. The evaluation using the Griffiths Scale showed a general quotient of 41.04 at four years, with homogeneous delays in various areas, and a general quotient of 38.3 at five years. At five years of age, he had short stature, cervicothoracic kyphosis, small feet, broad toes, and nonspecific facial dysmorphisms such as slight low-set ears and a prominent lower lip (Figure 1). He also had a symmetric intention tremor on manipulation [1,2].

In the etiological investigation, he underwent metabolic analyses, cranioencephalic magnetic resonance imaging, and visual and auditory assessments, which did not reveal any abnormalities. He was subsequently referred to a genetics consultation and underwent molecular analysis for Fragile X syndrome and array comparative genomic hybridization (array-CGH), which were both normal. Exome sequencing with analysis of copy number variations (CNV) identified a 300bp deletion involving the CUL4B gene. This deletion was classified as pathogenic and confirmed by quantitative polymerase chain reaction (qPCR), establishing the diagnosis of Cabezas syndrome (X-linked syndromic PDI type 15). The genetic testing of the parents revealed that the deletion occurred de novo [2-4].

## Discussion

Cabezas syndrome is a rare genetic pathology that has been reported in just over 100 cases in the medical literature [1,2]. The syndrome is characterized by ID and speech delay, both of which are typically evident from early childhood and remain disproportionately severe throughout the individual's life. In addition to these core features, the syndrome can also present with short stature, hypogonadism, abnormal gait, prominent lower lip, and tremor [1,4]. In actuality, the diagnosis of Cabezas syndrome becomes challenging only in the presence of alterations observed during objective examination, as observed in this case, rendering genetic testing a valuable diagnostic tool. In cases of Cabezas syndrome, the underlying genetic cause is typically a deletion of the CUL4B gene. However, this genetic abnormality is not always detected by standard diagnostic tests such as array-CGH platforms. In fact, in many cases, the diagnosis of Cabezas syndrome is only made through more advanced genetic testing techniques such as exome sequencing with CNV analysis. This particular phenomenon was documented in the medical case report in question, as well as in a singular similar case documented in 2020 [1-3].

Exome sequencing with CNV analysis has become an increasingly valuable tool in the diagnosis of genetic disorders associated with ID and GDD. This diagnostic approach has significantly increased the likelihood of identifying a genetic cause for these conditions, which can have important implications for patient care and genetic counseling [1-2].

Following the diagnosis, this patient was referred to educational support within the school setting, occupational and speech therapy interventions, as well as psychomotor and physical rehabilitation programs.

Early diagnosis of GDD and ID is critical to ensure that patients and their families receive appropriate guidance and follow-up care [1-3]. This is particularly important in the case of Cabezas syndrome, as the syndrome can have a significant impact on an individual's physical, cognitive, and social development. Early diagnosis can also help to identify potential therapeutic interventions that may help to alleviate symptoms and improve quality of life. Genetic counseling is another important aspect of the management of Cabezas syndrome. Individuals with this condition have a higher risk of transmitting the genetic abnormality on to their children, and genetic counseling can help families understand this risk and make informed decisions about family planning [2-4].

## Conclusions

In conclusion, this case highlights the importance of early neurodevelopmental assessment and the use of molecular techniques, such as exome sequencing with CNV analysis, in the etiological investigation of GDD and ID. The identification of the underlying genetic cause of these conditions not only provides a diagnosis but also allows for appropriate management and genetic counseling for the patient and their family. In the case of Cabezas syndrome, a rare genetic disorder with a high degree of clinical variability, genetic testing is crucial for diagnosis as the phenotype can overlap with other genetic syndromes. Furthermore, early detection of speech and language deficits in children is crucial as it can lead to early intervention and better long-term outcomes.

## References

[1] Okamoto N, Watanabe M, Naruto T (2017). Genome-first approach diagnosed Cabezas syndrome via novel CUL4B mutation detection. Hum Genome Var.

[2] Isidor B, Pichon O, Baron S, David A, Le Caignec C (2010). Deletion of the CUL4B gene in a boy with mental retardation, minor facial anomalies, short stature, hypogonadism, and ataxia. Am J Med Genet A.

[3] López M, Pérez-Grijalba V, García-Cobaleda I, Domínguez-Garrido E (2020). A 22.5 kb deletion in CUL4B causing Cabezas syndrome identified using CNV approach from WES data. Clin Case Rep.

[4] Cabezas DA, Slaugh R, Abidi F, Arena JF, Stevenson RE, Schwartz CE, Lubs HA (2000). A new X linked mental retardation (XLMR) syndrome with short stature, small testes, muscle wasting, and tremor localises to Xq24-q25. J Med Genet.

